# N-acetylcysteine attenuates cardiopulmonary bypass-induced lung injury in dogs

**DOI:** 10.1186/1749-8090-8-107

**Published:** 2013-04-22

**Authors:** Xianfeng Qu, Qianyu Li, Xiaofei Wang, Xiaoping Yang, Dongguo Wang

**Affiliations:** 1 Department of Anesthesiology, Taizhou Municipal Hospital, Taizhou, Zhejiang, 318000, P. R. China; 2 Clinical Laboratory, Taizhou Municipal Hospital, Taizhou, Zhejiang, 318000, P. R. China

**Keywords:** N-acetylcysteine, Cardiopulmonary bypass, Respiratory distress syndrome, Adult

## Abstract

**Background:**

Cardiopulmonary bypass (CPB) is usually associated with inflammatory response that leads to various degrees of organ dysfunction in multiple systems, including lung injury. Our previous study showed that transforming growth factor beta1 (TGFβ1) was involved in CPB-induced lung injury. N-acetylcysteine (NAC) is an antioxidant and is able to prevent CPB-induced pneumocyte apoptosis through scavenging radical. Therefore, we investigated whether NAC may attenuate CPB-induced lung injury by inhibiting TGFβ1 expression.

**Methods:**

Fifty-four 18 to 24-month-old mongrel dogs (15–16 kg) were randomly divided into control group, CPB group and NAC group (n = 18). Six dogs in each group were killed prior to, as well as 30 and 60 minutes after the operation (T0, T1 and T2). Lung injury was evaluated by hematoxylin and eosin (H&E) staining. Respiratory index (RI), oxygenation index (OI), malondialdehyde (MDA) content and superoxide dismutase (SOD) activity in the lung were determined at each time point. TGFβ1 expression was determined using real time RT-PCR and immunohistochemistry.

**Results:**

A serious lung injury was observed after CPB in dogs. RI and MDA content were increased significantly after CPB, whereas OI and SOD activity were decreased. H&E staining showed that NAC treatment obviously attenuated CPB-induced lung injury. NAC treatment upregulated OI and SOD activity and downregulated RI and MDA content in the lung tissues of dogs after CPB. Treatment with NAC significantly suppressed the TGFβ1 expression in the lung tissues at both mRNA and protein levels.

**Conclusion:**

Our results suggest that NAC is a potent agent against CPB-induced acute lung injury through inhibiting TGFβ1 expression.

## Background

Cardiopulmonary bypass (CPB), which is an essential technique for cardiac surgery, usually induces acute lung injury [1]. Lung injury clinically affects a small number of patients who undergo CPB. It is part of the CPB-induced organ dysfunction that may cause a collapsed lung and may develop into acute respiratory distress syndrome (ARDS) [1]. Therefore, CPB-induced acute lung injury is one of the main reasons for death after cardiac surgery [2].

Transforming growth factor beta1 (TGFβ1), a cytokine with complex functions, is able to regulate cell growth, proliferation and differentiation, and participate in the inflammatory response and tissue repair [3-6]. It has been reported that TGFβ1 participates in the endotoxin-induced acute lung injury through TGFβ1-Smad_2_ pathway [7]. TGFβ1 is abundantly expressed in the bronchoalveolar lavage fluid of the patients with ARDS during the early stage [8]. In our previous work, we also found the expression of TGFβ1 was upregulated in CPB-induced acute lung injury in dogs. It can directly increase the permeability of pulmonary artery endothelial cells and alveolar epithelial cells, resulting in the formation of pulmonary edema, the releasing of proinflammatory cytokines and thereby aggravating lung injury [9-11]. TGFβ1 also participates in the progression of lung fibrosis following injury [12]. Furthermore, TGFβ1 may aggravate the lung injury through increasing endothelial [13] and epithelial permeability [14] and decreasing ion and fluid transport [15,16]. In view of the important role of TGFβ1 in the development of acute lung injury, inhibition of TGFβ1 may be a potential therapeutic strategy for reducing CPB-induced acute lung injury.

N-acetylcysteine (NAC) is an antioxidant that could scavenge oxygen free radicals with protective effects on cells and microcirculation [17]. NAC is able to alleviate lung reperfusion injury induced by deep hypothermic circulatory arrest through scavenging oxygen free radicals [18]. It can reduce the pulmonary vascular resistance index and decrease the incidence of lung injury after off-pump coronary artery bypass surgery [19]. Interestingly, NAC can also prevent CPB-induced pneumocyte apoptosis through scavenging radical [20]. However, the relationship between NAC treatment and TGFβ1 expression in lung tissues has not been reported. In view of the important role of TGFβ1 during the progression of acute lung injury, the objective of this work was to investigate whether NAC could exert a protective effect on CPB-induced acute lung injury through inhibiting the expression of TGFβ1.

## Methods

### Animals and experiment design

Fifty-four 18 to 24-month-old mongrel dogs (15 ~ 16 kg) were purchased from the Experimental animal center of Zhejiang University. These dogs were randomly assigned into the control group, CPB group and NAC group (n = 18). Dogs were anesthetized with 2% sodium pentobarbital (i.p., 30 mg · kg^-1^), placed on a constant-temperature operating table and then endotracheal intubation was carried out. The animal was placed on an AH-II animal ventilator (Beijing science and Technology Development Company, Beijing, China) in the volume control mode. Initial ventilatory settings included: FIO_2_ 1.0, delivered tidal volume measured at the endotracheal tube 12 ml · kg^-1^. The respiratory rate (RR) was adjusted according to the result of blood gas analysis to maintain the PaCO2 between 35 and 40 mmHg. Mean arterial pressure (MAP) and central venous pressure (CVP) were monitored by an Intellivue MP50type multifunctional monitor (Philips, Holland) through placing femoral arterial and femoral venous catheters. Anesthesia was maintained with a continuous infusion of fentanyl (100 μg · kg^-1^ · min^-1^). Intermittent boluses of vecuronium bromide (0.1 mg · kg^-1^) was administered for neuromuscular blockade. The lung injury after CPB was induced according to the method reported by Williams *et al.*[21]. Briefly, after a median sternotomy, the pericardium was incised. After administration of heparin (3 mg · kg^-1^), cannulation was accomplished by placing an 8-French infant arterial cannula in the ascending aorta and a single 22-French venous cannula in the right atrium. Each dog was placed on conventional nonpulsatile CPB at a flow of (125 ml · kg^-1^ · min^-1^). Five minutes later, each animal was cooled for 10 mins to a nasopharyngeal temperature of 25°C. After cooling, the bypass flow was adjusted to 35 ml · kg^-1^ · min^-1^ for 60 mins, which achieved a mean arterial pressure of 35 ~45 mm Hg. During this period of low-flow CPB, the ventilator was set to deliver a constant positive airway pressure of 10 cm H_2_O with the FIO_2_ decreased to 0.21. After the 60-min period of low-flow CPB, the ventilator settings were adjusted to FIO_2_ 1.0, delivered tidal volume of 12 ml · kg^-1^. After rewarming to 37 °C for 20 mins, the animals were removed from CPB. Post CPB, adrenaline (1:1000, 0.1 ml · kg^-1^) and dopamine (10 μg · kg^-1^ · min^-1^) were administered for 60 min by a venous pump. Dogs in the control group underwent the same operation except for CPB. The dogs in NAC group were injected intravenously with NAC (Batch No. 2009081606, Sigma, USA) (150 mg · kg^-1^) through femoral vein before CPB according to the method in reference [22]. Then the dogs were continuously infused with NAC (20 mg · kg^-1^ · h^-1^) during the CPB process until the end of the experiment. The dogs in control group and CPB group were injected with equivalent normal saline. Six dogs in each group were sacrificed before CPB (T0), 30 min after CPB (T1) and 60 min after CPB (T2) and 2 ml arterial blood was obtained from each dog at each time point. Pulmonary tissues were sampled for future determination. The experiments were performed in accordance with the Helsinki Declaration of 1975 and approved by the Ethics Committee of Taizhou University Medical School. All the animals used in the study received humane care.

Respiratory index (RI) and oxygenation index (OI) were determined with the arterial blood using a GEMpremier ™3000 Blood gas analyzer (Instrumentation Laboratory, Ann Arbor, Michigan, USA).

Malondialdehyde (MDA) content and SOD activity of lung were measured using the thiobarbituric sodium method and xanthine oxidase method, respectively. Briefly, after the lung tissues were rinsed in pre-cool saline to remove the blood, the lung tissues were weighed and homogenized in 9 times the volume of homogenate medium (saline) using a Soniprep150 ultrasonic disintegrator (MSE company, United States). Then the homogenate was centrifuged at 3000 g for 12 min and supernatant was saved. The MDA content and SOD activity were measured with commercial kits from Nanjin Jiancheng Bioengineering Institute (Nanjing, China) using BeckmanDU800 (Beckman company, the United States of America) visible light analyzer.

### Hematoxylin and Eosin Staining

Pulmonary tissues were fixed with 4% paraformaldehyde, embedded in paraffin and stained with Hematoxylin and Eosin Staining (H&E) [23]. The images were obtained with a Leica DM1000 optical microscope and the pulmonary injury was scored by a blinded observer according to the following criteria [24]: alveolar congestion, hemorrhage, infiltration or aggregation of neutrophils in the airspace or vessel wall, and thickness of the alveolar wall. Each item was graded according to a 5-point scale: 0, minimal (little) damage; 1, mild damage; 2, moderate damage; 3, severe damage; and 4, maximum damage. Thus, minimum and maximum possible scores were 0 and 16, respectively.

### Immunohistochemistry

The expression of TGFβ1 was determined by immunohistochemical (IHC) staining with a commercial Strept Actividin-Biotin Complex (SABC) kit (Zhongshan Biological Technology, Beijing, China). IHC was carried out using a specific polyclonal anti- TGFβ1 antibody (1:150, Santa Cruz Biotech, Santa Cruz, CA, USA). The images were obtained with a Leica DM1000 optical microscope and analyzed by the image analysis system, Image-Pro Plus5.1.0.20 (Media Cybernetics, Silver Spring, MD, USA). Five unduplicated fields were selected in one slide.

### RNA extraction and semi-quantitative RT-PCR

Total RNA was extracted from pulmonary tissues with TRIzol reagent (Invitrogen, Carlsbad, CA, USA) according to the manual. The concentration and purity of total RNA were determined by a spectrophotometer (Eppendorf, Hamburg, Germany). Reverse transcription was performed using oligo (dT) primer RT mixtures with M-MLV (Promega, Madison, WI, USA) reverse transcriptase 200 U/20 μl, total RNA 1 μg/20 μl. cDNA was stored at −20°C. Primers used in the PCR reaction were as follows: β-actin, forward: 5′- GTA AAG ACC TCT ATG CCA ACA -3′, reverse: 5′- CTG GAA GGT GGA CAG TGA G -3′; TGFβ1: forward: 5′ –TAA TGG TGG ACC GCA ACA AC -3′, reverse: 5′- GTG AGC ACT GAA GCG AAA GC -3′. PCR reaction was performed with 2 μl of cDNA, 1 u Taq DNA Polymerase (Fermentas, MBI, USA) and 0.5 μmol/L of forward and reverse primers, for a total volume of 20 μl. Reactions were started with a polymerase activation step at 94°C for 1 min followed by 30 cycles of 94°C for 1 min, 54 C for 1 min and 72°C for 1 min, and elongation at 72°C for 10 min. The PCR products were electrophoresed on a 1.5% agarose gel in the presence of ethidium bromide, and absorbance was measured by an image analysis system. The ratio of targets to β-actin was used to evaluate the significance of the difference between the two groups.

### Statistical analysis

Statistical significance was determined using SPSS 13.0 for Windows. Measurement data are expressed as means ± S.D. Student’s t-test was performed to detect any differences between the two groups. Count data are expressed as median (range) and the difference in lung injury score between the two groups was tested using Kruskal-Wallis rank sum test. Differences were deemed significant if *P* < 0.05.

## Results

### NAC attenuated CPB-induced lung injury

**Figure 1:**
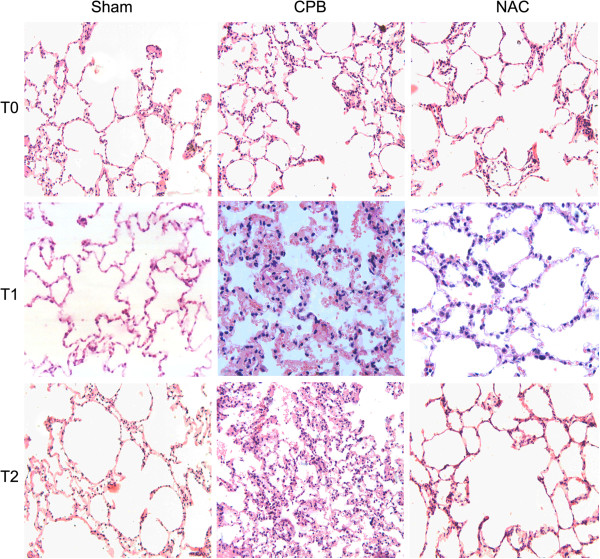
**HE staining of lung tissues.** HE staining was performed to detect the lung injury of dogs at three different time points. Six dogs in each group were examined (×200).

To investigate the effect of NAC on CPB-induced lung injury, H&E staining was performed. As shown in Figure 1, fine structure of lung was observed in the control group, without inflammatory cells infiltration. Smooth and complete pulmonary alveolar wall without bleeding and exudate in the pulmonary alveolar and interstitial was also observed in the lung tissues of control group (Figure 1). In the CPB group, disorder of the structure of lung tissues, and a large amount of fluid and a number of red blood cells in the pulmonary alveolar wall were observed. Marked hyperemia in the pulmonary alveolar wall, thickening of alveolar septa, edema in the pulmonary interstitium, extensive infiltration of inflammatory cells and focal atelectasis were also observed. With the treatment of NAC, the destruction on the lung structure was obviously reduced and little inflammatory cells were observed. The pulmonary injury score of NAC group was much lower than that of CPB group (Table 1).

**Table 1:** **The scores of lung injury at T2 time point (*n*** **= 6)**

**Group**	**Lung injury score**
Control	0.5 (0–1)
CPB	10.5 (7–14) ^a^
NAC	4 (2–5) ^b^

Data are expressed as median (range). Compared with control group, ^a^*P* < 0.05; compared with CPB group, ^b^*P* < 0.05.

### NAC regulated RI and OI post operation

There were no significant changes of RI and OI in control group post operation (Table 2). Compared with the values at T_0_ time point, RI values of CPB and NAC groups were increased, while OI values were decreased (*P* < 0.05) at T_1_ and T_2_ time points. The RI values of NAC group were lower and the OI values of NAC group were higher at T_1_ and T_2_ time points than those of CPB group, respectively.

**Table 2:** Effect of NAC on the levels of RI and OI

**Group**	**RI**			**OI**		
	**T0**	**T1**	**T2**	**T0**	**T1**	**T2**
Control	0.72 ± 0.03	0.74 ± 0.33	0.75 ± 0.43	422 ± 25	417 ± 27	420 ± 23
CPB	0.71 ± 0.04	4.71 ± 0.55 ^ab^	5.10 ± 0.13 ^ab^	420 ± 26	272 ± 25 ^ab^	220 ± 22 ^ab^
NAC	0.75 ± 0.04	0.79 ± 0.41 ^ac^	1.07 ± 0.63 ^ac^	425 ± 27	318 ± 26 ^abc^	306 ± 26 ^abc^

Blood samples were obtained from femoral artery at indicated time points and then RI and OI were analyzed. Data are expressed as means ± S.D (n = 6). Compared with T0 time point, ^a^*P* < 0.05; compared with control group, ^b^*P* < 0.05; compared with CPB group, ^c^*P* < 0.05.

### NAC regulated MDA and SOD post operation

The MDA levels and the SOD activity are considered as indicators of lipid peroxidation [25]. Therefore, we examined the MDA content and the SOD activity in the dogs’ lung tissues at different time points (Table 3). There were no obvious changes in MDA content and SOD activity in control group post the operation. The MDA content of lung tissues in CPB group was increased significantly after CPB, while treatment with NAC inhibited the increase of MDA content. In addition, there was a significant decrease of SOD activity in the CPB group at T1 and T2 time points compared with that at T0 time point. However, the decrease of SOD activity was significantly reversed by NAC treatment.

**Table 3:** Effect of NAC on the MDA and SOD levels at different time points

**Group**	**MDA (nmol/mg)**			**SOD (U/mg)**		
	**T0**	**T1**	**T2**	**T0**	**T1**	**T2**
Control	10.5 ± 1.0	10.8 ± 1.1	10.9 ± 1.2	154.7 ± 3. 9	150. 8 ± 4.8	151.69 ± 2.7
CPB	12.3 ± 1.5	25.8 ± 1.9 ^aabb^	24.9 ± 1.8 ^aabb^	153.6 ± 4.9	76.9 ± 3.8^aa^	82.2 ± 3.1^aa^
NAC	11.6 ± 1.0	16.5 ± 2.4 ^abbcc^	16.2 ± 2.1 ^abbcc^	150.7 ± 2.2	97.3 ± 2.4^aabbcc^	98.5 ± 3.6^aabbcc^

Data are expressed as means ± S.D (n = 6). Compared with T_0_ time point, ^a^*P* < 0.05, ^aa^*P* < 0.01; compared with control group, ^b^*P* < 0.05, ^bb^*P* < 0.01; compared with CPB group, ^c^*P* < 0.05, ^cc^*P* < 0.01.

### NAC inhibited the TGFβ_1_ expression in the lung tissues

**Figure 2:**
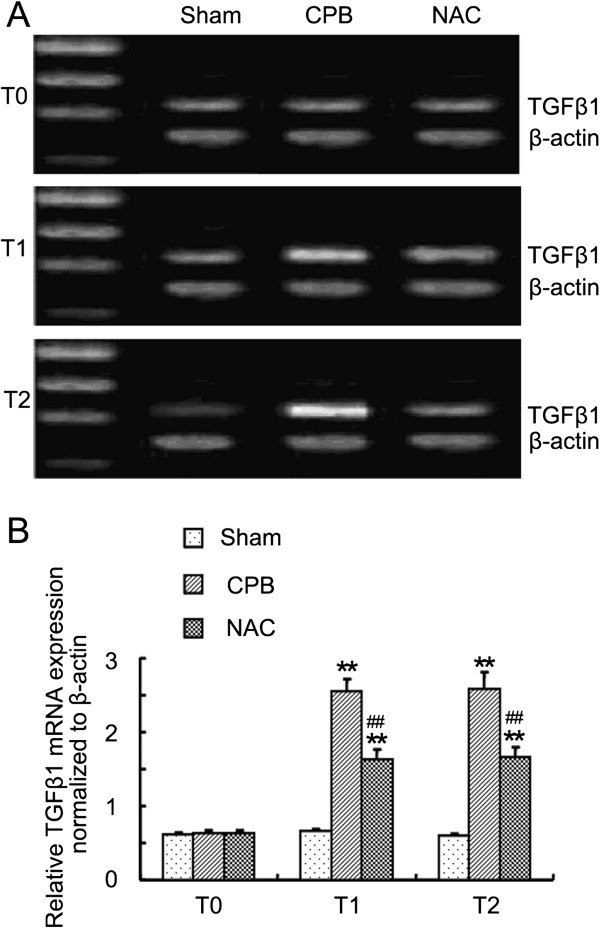
**Expression of TGFβ1 mRNA levels at each time point.** After the dogs were killed at each time points, total RNA of lung was isolated. Semi RT-PCR was performed to detect the TGFβ1 mRNA expression. **A**. Agarose gel electrophoresis of TGFβ1 and β-actin; **B**. Quantities for densitometric analysis of TGFβ1 mRNA. Data are expressed as mean ± S.D. (n = 6). ** *P* < 0.01, compared with control; ^##^*P* < 0.01, compared with CPB.

**Figure 3:**
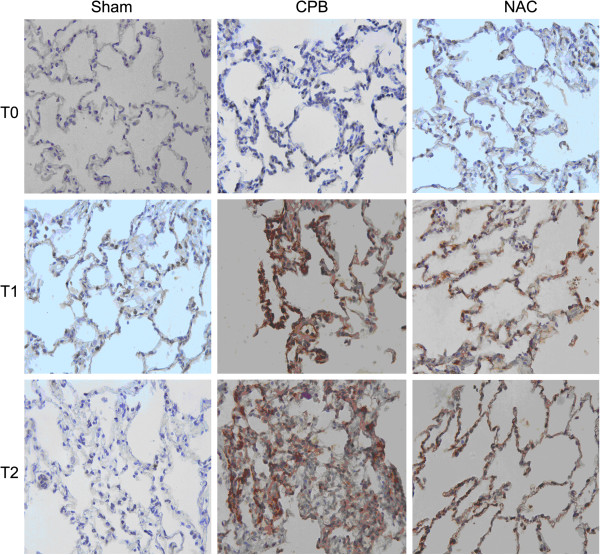
**Expression of TGFβ1 in the lung tissues at each time point.** Expression of TGFβ1 in lung tissues was determined by immunohistochemistry (×200).

To investigate the effect of NAC on the expression of TGFβ1 in lung tissues, we performed RT-PCR and immunohistochemistry. The expression of TGFβ1 mRNA increased significantly after CPB compared with those of control (Figure 2). TGFβ1 protein was also overexpressed in the lung tissues of dogs in CPB group after CPB as shown by immunohistochemical staining (Figure 3). Treatment with NAC significantly suppressed the TGFβ1 expression in the lung tissues at both mRNA and protein levels.

## Discussions

In the present study, we investigated the effect of NAC on CPB-induced acute lung injury. NAC reduced the pathological injury of the lung tissues in dogs. NAC treatment reversed CPB-induced upregulation of RI and MDA content, and downregulation of OI and SOD activity. CPB-induced expression of TGFβ1 in the lung of dogs was also inhibited by NAC treatment. The results showed that treatment with NAC attenuated the CPB-induced lung injury, indicating that NAC may be a potential drug for CPB-induced lung injury.

MDA is the end-products of lipid peroxidation and is considered as an indicator of the extent of lipid peroxidation, as well as the degree of tissue injury induced by oxygen free radicals. SOD is an oxygen free radical scavenger, reflecting the ability of scavenging oxygen free radicals. NAC, a micromolecule containing active hydrosulfide (−SH) with molecular weight of 163.29 g mol^-1^, could be quickly absorbed into the cells. As a sulfhydryl donor,it can scavenge oxygen free radicals and play a role in antioxidation directly. In addition, it could be converted to cysteine by stripping acetyl. Cysteine is the precursor of reduced glutathione hormone (GSH) which is able to scavenge oxygen free radicals [26]. Thus, NAC can also exert antioxidant effect indirectly. In the present study, our results showed that the MDA level was upregulated, while the SOD activity was downregulated in the lung tissues of dogs after CPB. In consistent with previous study [20], treatment with NAC significantly improved the lipid peroxidation injury in the lung tissues after CPB, indicating NAC may play a protective role in CPB-induced acute lung injury through its antioxidant effect.

TGFβ1 has been shown to be a critical mediator of acute lung injury [14]. The upregulation of TGFβ1 is usually related with the increase of the permeability of pulmonary endothelial monolayers and the permeability of alveolar epithelial monolayers [8]. The upregulation of TGFβ1 also leads to the decrease of sodium channel ENaC on the apical surface of alveolar epithelial cells [15], subsequently impairing the removal of salt and water from the alveolar lumen. Interestingly, it has also been reported that oxygen free radicals could activate pro-inflammatory nuclear factors, such as NF-κB and AP-1, and subsequently induce the expression of TGFβ1 [27-30]. Therefore, increased oxygen free radicals level in the injured lung tissues after CPB potentially lead to the upregulation of TGFβ1. Furthurmore, TGFβ1 could promote the release of oxygen free radicals to form the infernal circle of oxygen free radical-TGFβ1-lung injury. In the present study, we found NAC could not only inhibit the lipid peroxidation injury, but also inhibit the upregulation of TGFβ1 in the lung tissues of dogs after CPB, indicating that scavenging oxygen free radicals, attenuating lipid peroxidation, downregulating TGF*β*_1_ expression in the lung tissues and thereby blocking the infernal circle of oxygen free radical-TGFβ1-lung injury may be the mechanisms involved in the protective effect of NAC on CPB-induced acute lung injury in dogs.

## Conclusion

In conclusion, our results showed that NAC could alleviate the acute lung injury induced by CPB in dogs. The underlying mechanisms include scavenging oxygen free radicals and downregulating TGFβ1 expression in the lung tissues after CPB. Our results suggest that NAC is a potent agent against CPB-induced acute lung injury.

## Abbreviations

ARDS: Acute respiratory distress syndrome; CPB: Cardiopulmonary bypass; CVP: Central venous pressure; GSH: Glutathione hormone; H&E: Hematoxylin and eosin; IHC: Immunohistochemical; MDA: Malondialdehyde; MAP: Mean arterial pressure; NAC: N-acetylcysteine; OI: Oxygenation index; RI: Respiratory index; RR: Respiratory rate; SABC: Strept Actividin-Biotin Complex; SOD: Superoxide dismutase; TGFβ1: Transforming growth factor beta1.

## Competing interests

The authors declare that there is no conflict of interest that would prejudice the impartiality of this scientific work.

## Authors’ contributions

DW designed research; XQ, QL and XW performed research; XY analyzed data; DW and XW wrote the paper. All authors read and approved the final manuscript.
